# Chronic Disease Risk Factors Among American Indian/Alaska Native Women of Reproductive Age

**Published:** 2011-10-15

**Authors:** Pamela Amparo, Sherry L. Farr, Patricia M. Dietz

**Affiliations:** Centers for Disease Control and Prevention, Atlanta, Georgia; Centers for Disease Control and Prevention, Atlanta, Georgia; Division of Reproductive Health, National Center for Chronic Disease Prevention and Health Promotion, Centers for Disease Control and Prevention

## Abstract

**Introduction:**

The magnitude of chronic conditions and risk factors among American Indian/Alaska Native women of reproductive age is unknown. The objective of our study was to estimate this magnitude.

**Methods:**

We analyzed data for 2,821 American Indian/Alaska Native women and 105,664 non-Hispanic white women aged 18 to 44 years from the 2005 and 2007 Behavioral Risk Factor Surveillance System. We examined prevalence of high cholesterol, high blood pressure, diabetes, body mass index (kg/m^2^) ≥25.0, physical inactivity, smoking, excessive alcohol consumption, and frequent mental distress, and the cumulative number of these chronic conditions and risk factors (≥3, 2, 1, or 0). In a multivariable, multinomial logistic regression model, we examined whether American Indian/Alaska Native race was associated with the cumulative number of chronic conditions and risk factors.

**Results:**

American Indian/Alaska Native women, compared with white women, had significantly higher rates of high blood pressure, diabetes, obesity, smoking, and frequent mental distress. Of American Indian/Alaska Native women, 41% had 3 or more chronic conditions or risk factors compared with 27% of white women (χ^2^ *, P* < .001). After adjustment for income, education, and other demographic variables, American Indian/Alaska Native race was not associated with having either 1, 2, or 3 or more chronic conditions or risk factors.

**Conclusion:**

Three out of every 5 American Indian/Alaska Native women aged 18 to 44 years have 3 or more chronic conditions or risk factors. Improving economic status and education for AI/AN women could help eliminate disparities in health status.

## Introduction

Previous studies illustrate that American Indian/Alaska Native (AI/AN) women aged 18 years or older are disproportionately affected by chronic conditions (1-4). Compared with other adults, AI/ANs are more likely to smoke (1), be obese (1), and have higher rates of diabetes (4). In a 2001-2002 study looking at a limited number of chronic diseases and risk factors in US women aged 18 years or older, more than one-third of AI/AN women, in 2 AI/AN communities sampled, had 3 or more chronic diseases or risk factors, more than any other racial/ethnic minority group surveyed (3).

Reproductive-aged women with chronic medical conditions and related risk factors are at a greater risk not only for premature death and long-term illnesses but also for pregnancy complications. Smoking (5), diabetes (6,7), and hypertension (8) increase the risk of preterm delivery. Smoking (9), obesity (10), and diabetes (6,7) increase the risk of infant birth defects. Chronic diseases and risk factors are also associated with maternal complications during pregnancy such as gestational diabetes (11), gestational hypertension (6,7,12,13), and pre-eclampsia (6-8).

Many risk factors for chronic diseases are modifiable. Therefore, monitoring women in this age group for chronic conditions and risk factors can increase opportunities for early interventions to manage chronic conditions and help them adopt healthy behaviors to prevent or delay disease and improve future pregnancy outcomes. Although prevalence of certain chronic conditions has been assessed among all adult AI/AN women, the prevalence of multiple chronic diseases and risk factors among a nationally representative sample of AI/AN women of reproductive age has not previously been reported. In this study, we aimed to 1) estimate the prevalence of chronic conditions and risk factors among AI/AN and non-Hispanic white women, and 2) determine whether predictors of chronic conditions vary among these 2 groups.

## Methods

We analyzed data from the 2005 and 2007 Behavioral Risk Factor Surveillance System (BRFSS), a state-based, random-digit–dialed telephone survey of noninstitutionalized civilian adults aged 18 years or older throughout 50 states, the District of Columbia, and the US territories Puerto Rico, Guam, and the Virgin Islands. The 2005 and 2007 surveys were the most recently conducted surveys that included questions on cholesterol and blood pressure. The median state cooperation rate (percentage who were contacted and completed an interview) was 75.1% in 2005 and 72.1% in 2007, and the median response rate (percentage who were eligible and completed a survey) was 51.1% in 2005 and 50.6% in 2007. Response rates by race/ethnicity are not available in BRFSS. The Centers for Disease Control and Prevention's institutional review board did not require review of this study because it used de-identified, publicly available data.

We limited this report to nonpregnant AI/AN and non-Hispanic white women of reproductive age (18 to 44 y) because prevalence and associations with chronic disease risk factors may differ by pregnancy status, and too few pregnant women were surveyed to provide reliable individual estimates. Women self-reported information on age, marital status, education, annual income, and place of residence (urban or rural) in a telephone interview.

We analyzed 3 chronic conditions and 5 risk factors among women of reproductive age. The 3 chronic conditions of interest were high cholesterol, high blood pressure (chronic, only during pregnancy, prehypertensive or borderline high) and diabetes (chronic, only during pregnancy, prediabetes or borderline diabetes). Women answered a question about ever in their lifetime having had their cholesterol levels checked (yes, no) and 3 separate questions about ever in their lifetime having received a clinician diagnosis of high cholesterol (yes, no), high blood pressure (yes, prehypertensive or borderline high, only during pregnancy, no, don't know/not sure), and diabetes (yes, prediabetes or borderline diabetes, only during pregnancy, no, don't know/not sure). The 5 chronic disease risk factors of interest were being overweight or obese, not meeting national physical activity guidelines, current smoking, excessive alcohol consumption, and frequent mental distress. Overweight or obesity were defined as body mass index (BMI), calculated by using self-reported height and weight, of normal (<25.0 kg/m^2^), overweight (25.0-29.9 kg/m^2^), or obese (≥30.0 kg/m^2^). We defined meeting physical activity guidelines as doing 30 minutes of moderate-intensity physical activity on 5 or more days of the week or 20 minutes of vigorous-intensity physical activity on 3 or more days of the week as recommended by the American College of Sports Medicine and the American Heart Association (14). Current smoking was dichotomized as yes or no. We defined binge or heavy drinking as consuming 4 or more drinks on 1 occasion or more than 1 drink per day every day. Frequent mental distress was measured by answering the following validated question: "How many days during the past 30 days was your mental health not good?" (15). Women reporting 14 or more days were considered to be experiencing frequent mental distress. A composite variable was created by using the total number of the 8 chronic conditions and risk factors, categorized as 0, 1, 2, and 3 or more. Women also answered 3 questions on health care access and use: having health insurance, receipt of a routine checkup in the previous 12 months, and financial barriers to physician access in the previous 12 months.

Using χ^2^ tests, we examined differential distributions of demographic characteristics and chronic conditions and risk factors by race/ethnicity. On the basis of national guidelines that recommend women aged 20 years or older be tested for high cholesterol every 5 years (16), we examined the percentage of women aged 25 or older who had ever been tested for cholesterol. We also examined the association between AI/AN race and number of chronic conditions and risk factors reported (0, 1, 2, and ≥3) by using a multivariable multinomial logistic regression model, adjusting for other demographic characteristics. All analyses were performed using SUDAAN version 9.1 (RTI International, Research Triangle Park, North Carolina), including survey year as a sampling stratum, to account for the complex sample design; samples were weighted to produce unbiased national estimates. There were 2,821 AI/AN and 105,664 white women aged 18 to 44 surveyed in BRFSS in 2005 and 2007 and included in univariate analyses. Of the 108,485 women, 6,947 (6.4%) were missing information on 1 or more chronic conditions or risk factors, and another 8,639 (8.0%) were missing demographic information. These groups were not included in 1 or more of the multivariable models. Women excluded from the multivariable models because of missing information did not differ by race/ethnicity (*P* = .48) or year of survey (*P* = .62). However, excluded women were generally younger, unmarried, had lower educational status and income, and resided in rural areas (*P* < .001 for all).

## Results

Age, marital status, education, income, and place of residence were differentially distributed between AI/AN and white women (Table 1). Generally, AI/AN women were younger than white women and had fewer years of education and lower income. Fewer AI/AN women than white women were married or lived in an urban area.

Differences by race/ethnicity were found for having been tested for high blood cholesterol, for having clinical diagnoses of high blood pressure and diabetes, and for self-reported BMI, physical activity, smoking status, frequent mental distress, having health insurance, and limited access to care because of cost (Table 2). The most common chronic condition or risk factor for AI/AN women was being overweight or obese (53%), followed by not meeting national guidelines for physical activity (47%), and smoking (38%). Among white women, the most common conditions or risk factors were not meeting national guidelines for physical activity (45%), being overweight or obese (42%), and smoking (25%). Among women who had ever had their cholesterol checked, rates of high blood cholesterol were similar between AI/AN women and white women at 20%. However, 35% of AI/AN women aged 25 years or older had never had their blood cholesterol checked, compared to 24% of white women. Rates of gestational diabetes were similar for both groups. A lower percentage (74%) of AI/AN women had health insurance coverage compared to white women (86%). No difference between groups was seen in receipt of a routine checkup in the previous year; however, a higher percentage of AI/AN women (27%) compared to white women (17%) reported difficulty seeing a doctor in the last 12 months because of cost. Among AI/AN women and white women aged 25 years or older who had never had their cholesterol checked, 51% of AI/AN and 48% of white women had received a routine checkup in the previous 12 months (χ^2^, *P* = .47) (data not shown).

The total number of chronic conditions and risk factors differed between AI/AN and white women (χ^2^, *P* < .001) (Figure). Approximately 87% of AI/AN women had at least 1 chronic condition or risk factor, compared with 83% of white women. Among AI/AN women, 41% reported 3 or more chronic conditions or risk factors, compared to 27% of white women. The number of chronic conditions and risk factors among AI/AN and white women ranged from 0 to 8, with a mean of 2.2 for AI/AN and 1.7 for white women (*t* test, *P* < .001) (data not shown).

**Figure.**
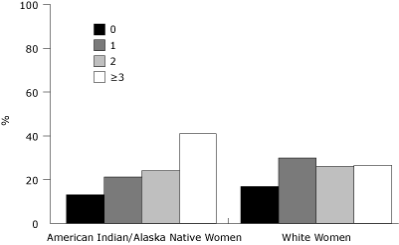
Number of chronic conditions and risk factors among American Indian/Alaska Native women and non-Hispanic white women aged 18 to 44 years, Behavioral Risk Factor Surveillance System, 2005 and 2007. Chronic conditions and risk factors are high cholesterol, high blood pressure (chronic, during pregnancy, prehypertensive or borderline high), diabetes (chronic, during pregnancy, prediabetes or borderline diabetes), being overweight or obese, physical inactivity, smoking, poor mental health, and binge or heavy drinking. All values for number of chronic conditions and risk factors are significant at *P* < .001, χ^2^ test.

**Table:** 

**Race**	**No. of conditions**			

	**0**	**1**	**2**	**≥3**
American Indian/Alaska Native women	13.2	21.2	24.3	41.4
White women	17.2	29.9	26.3	26.6

In the unadjusted logistic regression model, compared to white women, AI/AN women had higher odds of having 3 chronic conditions or risk factors but not 1 or 2 (Table 3). In the logistic regression model adjusted for all demographic characteristics, having 1, 2, or 3 or more chronic conditions or risk factors was not associated with AI/AN race. Income and education strongly confounded the relationship between AI/AN race and number of chronic conditions or risk factors. In a sensitivity analysis where we created an "unknown" level of income and included in the multivariable model an additional 8,313 women with missing data on income, the association between 1, 2, or 3 or more chronic disease conditions or risk factors and AI/AN race did not change.

## Discussion

The findings in this study illustrate the large burden of chronic conditions and associated risk factors among women of reproductive age and the disparity in chronic conditions and risk factors between AI/AN and white women. Although AI/AN and white women share the same top 3 chronic disease risk factors (being overweight or obese, doing less than the recommended physical activity, and smoking), the rates of being overweight or obese and smoking were 10 to 13 percentage points higher among AI/AN women. Additionally, the rates of diabetes and prediabetes were more than double those of white women, and rates of high blood pressure, borderline hypertension, and gestational hypertension were 46% to 80% higher among AI/AN women than white women. After adjusting for income and education, there was no association between AI/AN status and number of chronic conditions and risk factors. Therefore, the disparity in rates of chronic conditions and risk factors may be attributed to lower socioeconomic status and higher poverty among AI/AN women. This suggests that if economic status and education were improved for AI/AN women, then disparities in health status could be eliminated.

Pregnancy complications and poor birth outcomes from chronic conditions and risk factors are well known (5,17,18). These chronic conditions and risk factors also are established risk factors for future disease for the mother. Hypertension, high blood cholesterol, diabetes, obesity, and tobacco use are risk factors for cardiovascular disease. The overall AI/AN population has higher rates of diabetes mortality (19) and early cardiovascular death (20), which contribute to lower life expectancies compared to other race/ethnicities (19). Higher rates of chronic conditions and risk factors have been observed among AI/AN women compared with all US women aged 18 years or older (1,3,21), and the past decade's rates of obesity and smoking among AI/AN women were far from meeting *Healthy People 2010* goals (1). Our findings highlight the need for behavioral strategies such as diet, exercise, and smoking cessation among young AI/AN women. These strategies require screening and regular access to health care. In our analysis, 35% of AI/AN women aged 25 years or older had never had their blood cholesterol checked, which illustrates a need for screening and education and a potential underestimation of the prevalence of high cholesterol among young AI/AN women. Among these AI/AN women who had never had their cholesterol checked, more than half had had a routine checkup in the previous year, representing a missed opportunity for chronic disease screening and prevention. Access to health care was also lower for AI/AN women compared to white women. More than one-fourth of AI/AN women reported that cost prevented them from seeing a doctor in the previous year, a higher rate than that among white women. The Indian Health Service (IHS) and National Council of Urban Indian Health (NCUIH) provide health services to AI/ANs who live on or close to reservations and in urban areas and to those who may not have health insurance; these services may explain why there was no difference in the percentage of AI/AN and white women who received a routine checkup in the past year. However, AI/AN women reported more difficulty in seeing a doctor, indicating that cost and access to care are still issues for AI/AN women of reproductive age. This is consistent with another study that found that AI/AN populations had less insurance coverage and access to care than white women, and more than one-half of low-income uninsured AI/ANs did not have access to the IHS (22).

Four studies have shown similarly high rates of chronic conditions and risk factors among adult AI/AN women of all ages (1,3,21,23), but none, except for our study, adjusted for demographic characteristics such as income and education when examining the association between race/ethnicity and number of chronic conditions and risk factors. Our analysis was able to provide a more comprehensive profile of chronic conditions and risk factors among a sample of AI/AN women of reproductive age. Overall, decreasing poverty and improving education may decrease the disproportionate burden of disease among AI/AN populations. More specifically, targeting AI/AN women of reproductive age with prevention strategies and lifestyle modifications may reduce deaths and both short- and long-term illnesses and improve pregnancy outcomes. Chronic disease screening during reproductive health care visits may be an opportunity to reach these women as they interact with the health care system. In a nationally representative study of women aged 15 to 44 years, approximately three-fourths of the women had received sexual or reproductive health care services in the previous year (24). Additionally, 62% of women who receive care at a family planning center considered it their usual source of care (25).

This study has several limitations. Data were self-reported. Approximately 6% of women surveyed were missing data on 1 or more chronic conditions or risk factors, which may result in an underestimate of prevalences. Another 8% of women were missing data on demographic characteristics, 99% of whom were missing data on income. However, AI/AN status was not associated with missing information on chronic disease conditions and risk factors or income; therefore, the associations between AI/AN status and chronic conditions and risk factors should not be substantially affected by missing data. Including in the multivariable model women who were missing data on income did not affect odds ratio estimates for AI/AN women. Response rates for BRFSS have declined over the years; median response rates for 2005 and 2007 were approximately 50%. Additionally, BRFSS does not report rates by race/ethnicity. Two studies comparing BRFSS estimates to other national surveys, the National Health Interview Survey (26) and the National Health and Nutrition Examination Survey (27), found estimates to be similar and concluded that observed differences may be inconsequential. Additionally, BRFSS only reaches households with telephones and excludes homes without landlines or homes that use only cellular telephones, which may affect AIs to a greater extent than whites. People from households with only cellular telephones are more likely to report binge drinking, smoking, and being uninsured, and this disparity may be greatest for young and low-income adults (28). However, after adjusting for noncoverage of homes that use cellular telephones exclusively, the difference between cellular-only and landline users was less than 3 percentage points for all health indicators except binge drinking, which differed by 6 percentage points. Therefore, because of noncoverage of households whose members use only cellular telephones, we may have underestimated the prevalence of chronic conditions and risk factors among white and AI/AN women of reproductive age in the United States. It is likely that a larger number of AI/AN women live in rural areas than white women; therefore, the underestimation of chronic conditions and risk factors may be greater for AI/AN women than for white women. We also were unable to report data by tribal affiliation, which may conceal tribal or regional differences previously reported (21).

Our findings support the need to increase prevention efforts among AI/AN women of reproductive age. Screening for chronic conditions and referral to prevention programs may be incorporated into reproductive health visits while women are already seeking health care services. The IHS and NCUIH are comprehensive health care systems and health programs that may be well designed to accommodate this type of screening and referral process compared with other less comprehensive health care settings, for women who have access to them. However, for women without access, improvement in access to health care services is greatly needed. Screening, treating, and referring women of reproductive age with chronic conditions or related risk factors to comprehensive prevention programs may help prevent adverse pregnancy and long-term health outcomes for both women and their future children.

## Figures and Tables

**Table 1:** Demographic Characteristics of American Indian/Alaska Native (AI/AN) Women and Non-Hispanic White Women of Reproductive Age, Behavioral Risk Factor Surveillance System, 2005 and 2007^a^

Characteristic	AI/AN, Weighted % (95% CI)	Non-Hispanic White, Weighted % (95% CI)	*P* Value^b^
**Year**			
2005	47.5 (42.8-52.3)	50.2 (49.6-50.7)	.28
2007	52.5 (47.7-57.2)	49.8 (49.3-50.4)	
**Age, y**			
18-24	29.6 (24.4-35.3)	21.6 (21.1-22.2)	.01
25-29	14.9 (12.2-18.0)	14.5 (14.1-14.9)	
30-34	19.3 (16.2-22.8)	19.4 (19.0-19.8)	
35-39	17.0 (14.3-20.0)	20.1 (19.7-20.5)	
40-44	19.3 (16.4-22.7)	24.4 (24.0-24.9)	
**Marital status**			
Married	44.0 (39.3-48.7)	61.6 (61.0-62.1)	<.001
Divorced, separated, or widowed	14.1 (11.7-17.0)	10.0 (9.7-10.3)	
Never married	31.8 (27.2-36.9)	23.5 (23.0-24.1)	
Partner	10.1 (7.3-13.8)	4.9 (4.7-5.2)	
**Education**			
Less than high school graduate	14.9 (11.8-18.6)	5.7 (5.4-6.0)	<.001
High school graduate or equivalent	34.3 (29.7-39.1)	24.9 (24.4-25.3)	
Some college	29.9 (25.7-34.4)	30.8 (30.3-31.3)	
College graduate	21.0 (17.5-25.0)	38.7 (38.2-39.2)	
**Annual income, $**			
0-14,999	18.2 (13.9-23.3)	6.5 (6.2-6.8)	<.001
15,000-34,999	41.1 (36.1-46.3)	22.2 (21.7-22.7)	
35,000-49,999	15.5 (12.1-19.7)	16.6 (16.2-17.0)	
≥50,000	25.3 (21.5-29.4)	54.7 (54.2-55.3)	
**Place of residence**			
Urban	67.6 (63.6-71.3)	78.8 (78.4-79.1)	<.001
Rural	32.4 (28.7-36.4)	21.3 (20.9-21.6)	

Abbreviation: CI, confidence interval.

a 2,821 AI/AN women and 105,664 non-Hispanic white women aged 18-44 years.

b *P* values determined using  χ^2 ^test.

**Table 2:** Prevalence of Chronic Conditions, Associated Risk Factors, and Chronic Disease Screening Among American Indian/Alaska Native (AI/AN) and Non-Hispanic White Women of Reproductive Age, Behavioral Risk Factor Surveillance System, 2005 and 2007^a^

Condition or Risk Factor	AI/AN, Weighted % (95% CI)	Non-Hispanic White, Weighted % (95% CI)	*P* Value^b^
**Ever had blood cholesterol checked^c^**			
Yes	64.8 (60.3-69.1)	75.9 (75.4-76.4)	<.001
No or unsure	35.2 (30.9-39.7)	24.1 (23.6-24.6)	
**High blood cholesterol^d^**			
Yes	19.7 (16.0-24.0)	19.7 (19.2-20.2)	.76
No	80.1 (75.8-83.8)	80.0 (79.5-80.5)	
Unsure	0.2 (0.1-0.6)	0.3 (0.3-0.4)	
**High blood pressure**			
Yes^e^	12.0 (9.4-15.2)	8.2 (8.0-8.5)	.007
Only during pregnancy	4.7 (2.8-7.7)	3.1 (2.9-3.3)	
Prehypertensive or borderline high	0.9 (0.4-1.7)	0.5 (0.5-0.6)	
No	82.4 (78.5-85.7)	88.1 (87.8-88.5)	
Don't know or not sure	0.1 (0.0-0.4)	0.1 (0.0-0.1)	
**Diabetes status^f^**			
Yes^g^	5.4 (3.9-7.4)	2.2 (2.1-2.3)	<.001
Only during pregnancy	3.6 (2.2-5.9)	2.6 (2.4-2.7)	
Prediabetes or borderline diabetes	1.6 (0.9-2.7)	0.6 (0.5-0.7)	
No	89.5 (86.6-91.8)	94.6 (94.4-94.9)	
Don't know or not sure	0.0 (0.0-0.1)	0.0 (0.0-0.1)	
**Body mass index, kg/m^2^**			
Normal weight (≤24.9)	43.1 (38.2-48.1)	52.7 (52.2-53.2)	.001
Overweight (25.0-29.9)	27.0 (23.1-31.4)	23.2 (22.8-23.6)	
Obese (>30.0)	25.8 (22.5-29.5)	19.2 (18.8-19.6)	
Don't know, refused, or missing	4.1 (2.8-6.0)	4.9 (4.7-5.1)	
**Physical activity^h^**			
Yes; meets guidelines	53.0 (48.0-58.0)	55.0 (54.4-55.5)	.004
Yes; does not meet guidelines	34.7 (30.2-39.5)	37.8 (37.3-38.4)	
None	12.3 (9.0-16.6)	7.2 (6.9-7.5)	
**Smoking status**			
Current smoker	38.2 (33.6-43.0)	25.2 (24.7-25.6)	<.001
Never or former smoker	61.8 (57.0-66.4)	74.8 (74.4-75.3)	
**Alcohol consumption^i^**			
Heavy or binge drinker	21.3 (17.3-25.9)	18.1 (17.7-18.6)	.13
Moderate or never drinker	78.7 (74.1-82.7)	81.9 (81.4-82.3)	
**Frequent mental distress in last 30 days**			
Yes	19.6 (16.1-23.8)	13.1 (12.8-13.5)	<.001
No	80.4 (76.3-83.9)	86.9 (86.5-87.2)	
**Health insurance**			
Yes	74.2 (70.0-78.0)	85.7 (85.3-86.1)	<.001
No	25.8 (22.0-30.0)	14.3 (13.9-14.7)	
**Routine checkup within last year**			
Yes	66.9 (62.5-71.1)	64.9 (64.3-65.4)	.36
No	33.1 (28.9-37.5)	35.1 (34.6-35.7)	
**Difficulty seeing doctor in last 12 months because of cost**			
Yes	27.0 (23.0-31.4)	16.7 (16.3-17.1)	<.001
No	73.0 (68.6-77.0)	83.3 (82.9-83.7)	

Abbreviation: CI, confidence interval.

a 2,821 AI/AN and 105,664 non-Hispanic white women aged 18-44 years.

b P values determined using  χ^2 ^test.

c Per national guidelines (15), among 96,460 women aged ≥25 years.

d Among 76,975 women who had ever had their blood cholesterol checked.

e Excluding pregnancy-induced high blood pressure.

f Diabetes defined as answering yes to the question "Have you ever been told by a doctor that you have diabetes?"

g Excluding pregnancy-induced diabetes.

h Based on American College of Sports Medicine and American Heart Association recommendations (14) of 30 minutes of moderate-intensity physical activity on 5 days or more of the week or 20 minutes of vigorous-intensity physical activity on 3 days or more of the week.

i Binge/heavy drinking is defined as 4 or more drinks on 1 occasion or more than 1 drink per day every day.

**Table 3:** Comparison of the Number of Chronic Conditions or Risk Factors^a^ Reported by American Indian/Alaska Native Women and Non-Hispanic White Women of Reproductive Age, Behavioral Risk Factor Surveillance System, 2005 and 2007

**No. of Chronic Conditions**	Unadjusted OR (95% CI)^b^	AOR (95% CI), Full Model^c^
≥3 vs 0	2.0 (1.4-3.1)	1.3 (0.8-2.0)
2 vs 0	1.2 (0.8-1.8)	0.9 (0.6-1.4)
1 vs 0	0.9 (0.6-1.5)	0.8 (0.5-1.2)

Abbreviations: OR, odds ratio; CI, confidence interval; AOR, adjusted odds ratio.

a Based on reported presence of high cholesterol, high blood pressure (chronic, during pregnancy, prehypertensive or borderline high), diabetes (chronic, during pregnancy, prediabetes or borderline diabetes), being overweight or obese, physical inactivity, smoking, frequent mental distress, or heavy or binge drinking.

b n = 101,538 women who were not missing information on chronic conditions or risk factors.

c n = 92,899 women who were not missing information on chronic conditions, risk factors, and demographic variables that were included in model. Adjusted for survey year, age, marital status, place of residence (urban or rural), education, and annual income.
